# BPD Following Preterm Birth: A Model for Chronic Lung Disease and a Substrate for ARDS in Childhood

**DOI:** 10.3389/fped.2016.00060

**Published:** 2016-06-15

**Authors:** Anita Bhandari, Christopher Carroll, Vineet Bhandari

**Affiliations:** ^1^Division of Pediatric Pulmonology, Connecticut Children’s Medical Center, University of Connecticut School of Medicine, Hartford, CT, USA; ^2^Division of Pediatric Critical Care Medicine, Connecticut Children’s Medical Center, University of Connecticut School of Medicine, Hartford, CT, USA; ^3^Section of Neonatology, St. Christopher’s Hospital for Children, Drexel University College of Medicine, Philadelphia, PA, USA

**Keywords:** BPD, pediatric ARDS, lung diseases, biomarkers, lung development

## Abstract

It has been suggested that pediatric acute respiratory distress syndrome (PARDS) may be a different entity, vis-à-vis adult acute respiratory distress syndrome (ARDS), based on its epidemiology and outcomes. A more pediatric-specific definition of PARDS to include the subgroup of patients with underlying lung (and heart) disease has been proposed. Epidemiological data suggest that up to 13% of the children with ARDS have a history of prematurity and/or underlying chronic lung disease. However, the specific contribution of bronchopulmonary dysplasia (BPD), the most common chronic lung disease in infants, to the development of PARDS is not known. BPD leads to damaged lungs with long-term consequences secondary to disordered growth and immune function. These damaged lungs could potentially act as a substrate, which given the appropriate noxious stimuli, can predispose a child to PARDS. Interestingly, similar biomarkers [KL-6, interleukin (IL)-6, IL-8, sICAM-1, angiopoietin-2, and matrix metalloproteinase-8 and -9] of pulmonary injury have been associated both with BPD and ARDS. Recognition of a unique pattern of clinical symptomatology and/or outcomes of PARDS, if present, could potentially be useful for investigating targeted therapeutic interventions.

## Introduction

Bronchopulmonary dysplasia (BPD) is the most common chronic respiratory disease in infants and is a devastating condition that disrupts the developmental program of the lung secondary to preterm birth. In 2013, one in nine (11.4% of all live births) babies was born at <37 weeks gestation (1). Babies born at <32 weeks gestation account for 1.9% of live births, resulting in more than 75,000 babies admitted to neonatal intensive care units (NICUs) each year (1). Of all births in the US, 1.4% (55,548/year) are of very low birth weight (VLBW; <1500 g) infants annually (1), of whom 15,000 develop BPD (2). In the US, BPD is the leading cause of chronic lung disease in babies and the third leading cause in children (2, 3).

Despite many advances in neonatal ventilation techniques, widespread use of surfactant and antenatal corticosteroids, the incidence of BPD has remained the same (4) or even increased slightly (5, 6). The emotional and financial burden of caring for babies with BPD is significant. Babies with BPD require intensive hospital care for an average of 120 days. In 2009, the presence of BPD in an infant increased mean direct hospital costs (not including physician fees) in the NICU by ~$60,000 (7). Management of BPD takes a considerable toll on health services. Among preterm infants, the single costliest complication of hospitalization during infancy is BPD, with an average cost per discharge of $116,000 (8). Additionally, BPD is associated with significant pulmonary and neurodevelopmental sequelae that continue to have health ramifications into adulthood (5, 9, 10). Home oxygen therapy, when required, averages 92 days. Up to 50% of babies with BPD require readmission to the hospital for lower respiratory tract illness in the first year of life. Median medical costs for home care after discharge is $8100 per child; if the child requires hospitalization, the cost increases up to $50,000. The overall costs of treating babies with BPD in the US are estimated to be $2.4 billion. This amount is second only to the costs for treating asthma and far exceeds the costs for treating cystic fibrosis.

## Pathogenesis of BPD

There are five distinct stages in lung development, namely, embryonic, pseudoglandular, canalicular, saccular, and alveolar (11, 12). Preterm infants who are predisposed to developing BPD are born in the late canalicular or early saccular stage of lung development. In the late canalicular stage, there is development of the primitive alveoli and the alveolar capillary barrier, and the differentiation of Type I and Type II pneumocytes (13). In the early saccular stage, surfactant production starts along with pulmonary vascularization and enlargement of terminal airways (11, 12, 14, 15). The lungs complete their development after birth, which continues up to 8 years of age (16). Alveolar sacs are formed by secondary septation of alveolar ducts (13). BPD is caused due to an interaction between genetic and environmental factors (hyperoxia, invasive mechanical ventilation, and sepsis) (5, 17–19).

The foundation of BPD is the immature lung, which may or may not be surfactant deficient. Genetic predisposition probably accounts for 50–80% of the susceptibility to BPD (20, 21). The genetically predisposed immature lung is exposed to noxious external factors: pre- and postnatal infections, hyperoxia, and ventilator-induced barotrauma, volutrauma, or atelectotrauma, which initiates an inflammatory cascade involving inflammatory cells and signaling *via* various cytokines, chemokines, and growth factors. This, in turn, activates the cell death pathways. Damage to the developing lung is followed by resolution of injury to close to normal lung architecture or repair (19). The “old” or “classical” BPD occurred in the pre-surfactant days where invasive ventilation practices led to significant lung injury characterized by a severe fibroproliferative response along with airway injury and alveolar growth abnormalities (22). In the post-surfactant, “gentler” ventilation era in the more immature lung, this reparative state of the lung is characterized by fewer and larger simplified alveoli, along with dysmorphic vasculature, leading to the description of impaired alveolarization and dysregulated vascularization – the pulmonary phenotype of “new” BPD (5, 18, 22–24).

It is important to highlight the fact that for BPD to occur, it requires the known environmental factors to be exposed to the immature lung for a sustained duration, resulting in persistent inflammation (13). While markers of the early inflammatory response (cells, cytokines) may not be detectable after the prolonged exposure to the damaging environmental agents, the initial cascade of the signaling pathways of the inflammatory process/immune response does eventually lead to permanent defects of structure and function in the BPD lungs (13). Anatomical and functional pulmonary deficits in children and adults who had BPD during infancy have been well documented (25–27).

## Epidemiology of Pulmonary Outcomes up to Childhood

High resolution chest CT scans (HRCT) in survivors of old BPD and new BPD both show persistent radiological abnormalities with structural changes (28–33). The extent of the structural abnormality has been found to be associated with abnormal pulmonary function tests (28, 30, 31). In younger patients with new BPD, Mahut et al. have reported that the extent of structural abnormalities is correlated with duration of mechanical ventilation and supplemental oxygen (32).

Structural changes in lungs of BPD survivors based on HRCT are more marked in the peripheral lung in both old and new BPD survivors (34).

As one would expect, a stormy perinatal period leads to lasting effects. Survivors of BPD continue to have a greater respiratory morbidity and need for respiratory medications into young adulthood. They are also more likely to have chest wall deformities and a diagnosis of asthma during childhood (35). They have greater rates of hospitalization especially in the first 2 years of life as compared with infants born full term (36, 37). Although rates of hospitalization decrease as these children get older, long-term lung function abnormalities persist. Patients with old BPD had markedly abnormal lung function during late adolescence and 68% of which had evidence of airway obstruction. There was a marked decrease in forced expiratory volume in 1 s (FEV1), forced vital capacity (FVC), and the average forced expiratory flow during the mid (25–75%) portion of the FVC (FEF 25–75%) with evidence of gas trapping in survivors of BPD, as compared with age matched controls who were term and preterm infants without BPD (38). In adolescent patients (mean age 17.7 years) who were born preterm, Halvorsen et al. reported a lower FEV1 when compared those born at term (39). Doyle et al. also reported lung function tests of BPD survivors at a mean age of 18.9 years. They found a significant decrease in FEV1 and FEV1/FVC ratios in patients with a history of BPD, compared with those with no BPD (40). Patterns of lung function abnormalities were similar in survivors of old and new BPD. In patients with new BPD, Fawke et al. found that in over half of the cohort of children born at <26 weeks of gestation that they followed had abnormal spirometry at 11 years of age (35). Others too have reported small airway abnormalities and diffusion abnormalities in infants and young children with BPD (41–43) and persistent abnormalities into late childhood (40, 44–47). BPD survivors with persistent airflow obstruction are less likely to respond to bronchodilators as compared with patients with asthma with a similar degree of obstruction, suggesting that some of the small airway dysfunction seen in BPD survivors may be related to structural change rather than eosinophilic inflammation (48). It is difficult to directly compare outcomes in patients with old BPD with new BPD due to the lack of conformity in the definition of BPD between the two eras since relatively more immature babies are surviving in the post-surfactant era as compared with the pre-surfactant era. It is important to mention here that, while not the focus of this article, significant abnormalities in pulmonary function tests in infants with BPD extend well into late adolescence and adulthood (31, 38–40, 49–52). On the other hand, in adult survivors of acute respiratory distress syndrome (ARDS) (median age 44 years), pulmonary function has been shown to improve over 5 years with the majority of patients having normal and near normal lung function (53).

Thus, a damaged lung with disordered growth and immune function could potentially act as a substrate, given the appropriate noxious stimuli, to predispose a child to ARDS. However, until recently, the definitions for ARDS were not well suited for children. This may have led to an under diagnosis of ARDS in children with underlying lung disease.

## Epidemiology of ARDS/PARDS

When first described by Ashbaugh et al. in the year 1967 (54), adult respiratory distress syndrome was defined as respiratory failure with the following three criteria, such as (1) PaO_2_/FiO_2_ ratio of <300 mm of Hg, (2) diffuse bilateral infiltrates on chest radiograph, and (3) an identifiable insult within 7 days of developing a compromise in oxygenation and a well preserved left ventricular function. The terminology of “respiratory distress syndrome” was coined as a reference to a similar neonatal condition. In 1994, recognizing that the adult respiratory distress syndrome occurs in both children and adults, the American-European Consensus Conference (AECC) changed the nomenclature from *adult* respiratory distress syndrome to *acute* respiratory distress syndrome (ARDS) and defined it as a syndrome of inflammation and increased permeability in the lungs that is associated with a constellation of clinical, radiological, and physiological abnormalities that cannot explain but coexist with left atrial or pulmonary capillary hypertension (55).

In clinical practice, the adult criteria were used for more than a decade in the pediatric population. However, there were limitations in the use of this definition in children, including differences in triggering disease states, epidemiology, and outcomes, and technical problems of assessing oxygenation in children without arterial blood gas measurements. The Berlin definition has addressed some of the limitations of the AECC definition (56) better, in terms of validity, for predicting mortality with ARDS, but still lacked pediatric perspective. Pediatric acute respiratory distress syndrome (PARDS) may be a different entity specifically based on its epidemiology and outcomes. A group of experts have proposed a more pediatric-specific definition of PARDS to account for differences in adults and children (57). Authors propose the following changes in the (a) simplification of chest imaging criteria to eliminate presence of bilateral infiltrates, (b) use of pulse oximetry-based criteria when PaO_2_ is not available, and (c) inclusion of oxygenation index instead of PaO_2_/FiO_2_ with a minimum positive end expiratory pressure for invasively ventilated patients and specific inclusion of children with underlying lung and heart disease.

Pediatric acute respiratory distress syndrome is less common as compared with ARDS with an incidence of 2.0–12.8 children per 100,000 patient years with a lower morality in PARDS as compared with ARDS (58–62). The incidence of PARDS is likely underestimated, especially in patients with a preexisting lung disease. In a child with underlying lung disease (such as BPD), it can be difficult to make a diagnosis of ARDS given the Berlin definition. An acutely ill child with underlying lung disease is often diagnosed with an exacerbation of their underlying disease rather than ARDS, possibly leading to an under diagnosis and underestimation of PARDS (59). The revised definition of PARDS to specifically include the subgroup of pediatric patients with underlying lung and heart disease in the definition of PARDS may improve the recognition of this condition.

## Pathogenesis of ARDS/PARDS

Similar to adults, the most common causes of ARDS in the pediatric age group are underlying pneumonia or viral illness (58, 63). Sepsis, aspiration, and trauma can also cause ARDS in children, but occur less frequently in children compared with adults. A significant proportion of patients of PARDS have underlying chronic comorbidities. Of these, underlying chronic lung disease is one of the commonest comorbidity reported in pediatric patients who develop ARDS/acute lung injury (ALI) (62). Prematurity has been reported in 13% of the children with ARDS (45), and underlying chronic lung disease has been reported to occur in 11–13% (62, 63). However, in some studies of ARDS in children, patients with underlying hypoxic lung disease were excluded (58). Nevertheless, children with BPD are at risk for developing ARDS, especially if they have a history of severe BPD that is often associated with other comorbidities. These include swallow dysfunction and neurodevelopmental delay, which puts them at risk for impaired airway clearance and aspiration pneumonia, increasing the risk of recurrent pulmonary infections. Therefore, it becomes difficult to determine if patients with BPD have a flare up of their underlying condition or have ARDS triggered by infection, aspiration, or other pulmonary insults. This may also affect the management of a patient with acute exacerbation of underlying BPD, which may be significantly different from a patient with ARDS with underlying BPD; for example, the use of corticosteroids would probably be more likely in the former category of patients. In addition, pulmonary vascular disease and coexistent pulmonary hypertension play a role in the morbidity of infants with BPD presenting with respiratory failure. Pulmonary vasodilatory therapies, such as inhaled nitric oxide (iNO), may help in these circumstances (2). This variability in reported data presents challenges in sorting out whether past history of BPD increases the risk of development of ARDS or affects outcomes in this population.

Despite multiple chronic comorbidities in those with PARDS, presence of underlying immunodeficiency is the only comorbidity associated with poor outcome (64, 65). Others have reported that non-pulmonary sepsis is associated with increased mortality in patients with ALI (62, 65). However, most epidemiological studies of ARDS do not list specific diagnoses as comorbidities or underlying conditions and, instead, describe organ system involvement more broadly. Since BPD is one of the commonest forms of preexisting lung disease, it may be reasonable to extrapolate that there is no clear associated increase in risk of death with PARDS in patients with BPD. However, it is important to clarify that there is no specific data to support or refute the extrapolated statement.

Some studies have suggested a predominance of Caucasian infants being predisposed to BPD (66); this finding has not been consistent (67). Given the significant genetic susceptibility to BPD, it very likely that if specific single nucleotide polymorphisms (SNPs) occur more commonly in a particular race/ethnic group, that would be more important for predisposing a particular race/ethnic group towards developing BPD (68, 69). It is important to mention here that race/ethnicity has been noted to impact on lung function and utilization of respiratory medications in infants born preterm and/or with BPD (70–72). African-American adults are more likely to have a more severe disease and greater mortality with ARDS. Hispanic adults also have higher mortality rates independent of severity of disease (73). There is no reported race bias in terms of mortality in PARDS. Male gender has also been shown to increase the risk of developing BPD (74, 75). However, similar to the data reported in adults with ARDS, in PARDS, there is no difference in mortality rates based on gender (58, 62, 63, 76, 77).

## Outcomes of PARDS

There is significant mortality associated with PARDS; however, these mortality rates appear to be lower than that in adults, ranging from 15 to 50% mortality in children compared with 35 to 45% mortality in adults (60, 62). There is, however, a wide range in the mortality range of the cohorts of children with ARDS (47). This wide range in mortality assessment may be due to differences in the comorbid or underlying conditions in the children in these relatively small cohorts or may be due to improvements in care over time (47).

## Biomarkers in BPD and ARDS

Theoretically, biomarkers can be used to identify at risk population, predict severity of illness, target therapy, and predict prognosis.

Various biomarkers detected in different biological fluids have been proposed for early identification of infants predisposed to BPD (3, 78). The majority of the studies have been conducted using blood, urine, or tracheal aspirate (TA) samples (3, 78). Among these that have been implicated both in BPD and ARDS (*vide infra*) are the following.

KL-6 (a lung injury marker) was increased in infants in the umbilical cord blood samples in infants who went on to develop BPD (*n* = 50 vs. non-BPD *n* = 24) (79) and was also useful as a predictor of moderate/severe BPD at 1 week of postnatal life (80). Combining results from all models, BPD/death (*n* = 606 out of 1062) was associated with higher levels of interleukin (IL)-6 and -8 in blood samples collected at various timepoints during the infants’ stay in the NICU (81).

Among the cytokines, it is important to note consistent results with increased blood levels of IL-6 and -8 have been associated with BPD (3). This is supported by increased concentrations of these cytokines in TA obtained from human infants developing BPD (3). Among other pulmonary biomarkers of BPD, two independent cohorts have reported increased TA levels of angiopoietin-2 (Ang-2) to be associated with increased risk of BPD and/or death (82, 83).

Soluble intercellular adhesion molecule-1 (sICAM-1) is increased in TA samples in the first week of life in infants developing BPD (77, 84). Increased levels of matrix metalloproteinase (MMP)-8 and -9 have been reported in infants developing BPD (18, 77, 84).

In a systematic meta-analysis of plasma-derived biomarkers associated with ARDS, Terpstra et al. reported ranking of plasma biomarkers for ARDS according to the strength of association with both diagnosis and mortality of ARDS (85). Authors found that KL-6, lactic dehydrogenase, soluble RAGE, and von Willebrand factor are strongly associated with the development of ARDS in at-risk patients. They also concluded that mortality with ARDS was most strongly associated with IL-4, IL-2, Ang-2, and KL-6. Increased plasma levels of transferrin and protein C were associated with decreased odds for both development of ARDS and mortality with ARDS (85). Studies examining the role of inflammatory biomarkers in PARDS are far fewer than in ARDS (86). Serum IL-8 and IL-6 have both been shown to be elevated in patients with PARDS (86). While elevated levels of plasma sICAM-1 on days 1 and 2 of ALI were associated with increased risk of death and prolonged mechanical ventilation, Flori et al. noted that when combined with a physiological index of oxygenation defect (PaO_2_/FiO_2_), it further strengthened the predicted value risk of death in PARDS (87). Higher TA MMP-8 and MMP-9 levels in patients with PARDS have been shown to correlate with increased risk of death and prolonged mechanical ventilation (87, 88). The biomarkers have been categorized into markers of pulmonary epithelial or endothelial ling injury and summarized in Table 1.

**Table 1:** **Biomarkers of lung injury that are similar in BPD and ARDS**.

Epithelial cells	Endothelial cells
Angiopoietin-2^a^	Angiopoietin-2^a^
KL-6	sICAM-1
IL-6	
IL-8	
MMP-8	
MMP-9	

*^a^Can be made/released by both epithelial and endothelial cells*.

*IL, interleukin; MMP, matrix metalloproteinase; sICAM, soluble intercellular adhesion molecule*.

It is unclear what causes the difference in outcomes of pediatric patients, as compared with adults with ARDS. It has been suggested that in pediatric patients the stage of lung development at the time of the insult may be one cause of this outcome difference. This may pertain especially to younger children. It is known that alveolar development is a primarily postnatal process which starts at ~36 weeks of gestation and continues in the postnatal period. Maximal alveolar growth is completed by 24 months of age, although there may be some growth that occurs for up to 8 years. Although this has not been researched adequately, in a child where alveolar growth is still not complete, ARDS may lead to a different pathology, thus affecting not only the course of the disease but also the long-term outcome.

Acute respiratory distress syndrome is primarily an inflammatory response to injury. There is evidence to suggest that children have a unique inflammatory response to injury, which confers protection from development of multi-organ failure, although underlying mechanisms are not known (89). It is fair to say that the child’s inherent immune response may have some role to play in the difference in the course and outcomes of PARDS.

## Lack of Data and Need for Future Research

Pediatric acute respiratory distress syndrome is less common than ARDS; furthermore, epidemiological studies of PARDS do not provide specific diagnoses as comorbidities or underlying conditions. This limits our understanding of how underlying lung disease (specifically BPD) may affect the pattern of clinical presentation and/or outcomes of PARDS. Newer criteria of PARDS have been proposed recently, which we hope will help better understand the contribution of underlying chronic lung disease to PARDS. Proper categorization and definition of ARDS in patients with underlying disease, such as BPD, are also important to guide clinical therapy. This review highlights many questions that remain unanswered and underscores the need for future multidisciplinary collaboration to study the long-term outcome of BPD and as a substrate for PARDS.

## Summary and Conclusion

Chronic pulmonary disease is a commonly seen comorbidity in patients with PARDS. There is no clear data to suggest an association of BPD with increased probability of developing ARDS, neither is there any suggestion that underlying lung disease portends a worse outcome with ARDS. However, similar biomarkers (KL-6, IL-6, IL-8, sICAM-1, Ang-2, and MMP-8 and -9) of pulmonary injury have been associated both with BPD and ARDS. Current practice where pediatric patients are more likely to be diagnosed with their underlying disease rather than PARDS underestimates the true incidence of ARDS, and possibly the effect of underlying chronic lung disease. Future research incorporating listing BPD as a specific underlying diagnosis would assist in recognition of a unique pattern (if present) of clinical presentation and/or outcomes of PARDS, which, in turn, could potentially set the stage for investigating targeted therapeutic interventions.

## Author Contributions

AB: concept, design, interpretation, analysis, revision for critical intellectual content, and final approval of draft. CC: interpretation, analysis, revision for critical intellectual content, and final approval of draft. VB: concept, design, initial draft, interpretation, analysis, revision for critical intellectual content, and final approval of draft.

## Conflict of Interest Statement

The authors declare that the research was conducted in the absence of any commercial or financial relationships that could be construed as a potential conflict of interest.
